# Evaluation and Optimization of Microdrop Digital PCR for Detection of Serotype A and B *Clostridium botulinum*

**DOI:** 10.3389/fmicb.2022.860992

**Published:** 2022-05-09

**Authors:** Pengya Gao, Changde Wu, Jin Zhang, Shuping Wang, Ying Huang, Yinping Dong, Tingting Liu, Changyun Ye, Xuefang Xu, Wenwen Xin

**Affiliations:** ^1^State Key Laboratory for Infectious Disease Prevention and Control and National Institute for Communicable Diseases Control and Prevention, Chinese Center for Disease Control and Prevention, Beijing, China; ^2^College of Animal Science and Veterinary Medicine, Shenyang Agricultural University, Shenyang, China; ^3^Criminal Investigation School, People's Public Security University of China, Beijing, China; ^4^NHC Key Laboratory of Food Safety Risk Assessment, China National Center for Food Safety Risk Assessment, Beijing, China; ^5^State Key Laboratory of Pathogen and Biosecurity, Institute of Microbiology and Epidemiology, Academy of Military Medical Sciences, Beijing, China

**Keywords:** *Clostridium botulinum*, droplet digital PCR, rapid clinical diagnosis, neurotoxin, q-PCR

## Abstract

*Clostridium botulinum* is the causative pathogen of botulism. Laboratory detection of *C. botulinum* is essential for clinical therapy treatment of botulism due to the difficulty in diagnosis, especially in infant botulism. The extreme toxicity of botulinum neurotoxin (BoNT) requires a sensitive detection method. Due to the detection limit of real-time quantitative PCR (q-PCR), a more sensitive detection method, micro-drop digital PCR (ddPCR) was applied in *C. botulinum* main serotypes A and B. The following performance criteria were evaluated by ddPCR: analytical sensitivity; repeatability; and diagnostic specificity. The limit of detection (LOD) was 0.84 and 0.88 copies/μl for BoNT A and B genes, respectively, by ddPCR with high specificity, compared to 5.04×10^2^ and 6.91×10^2^ copies/μl by q-PCR. It was increased 10 times compared with q-PCR in spiked stool samples. This improvement in sensitivity was especially important in clinical samples as more positive samples were detected by digital PCR compared with q-PCR. Meanwhile, enrichment time for low bacteria content samples was shortened by four hours both in serotypes A and B *C. botulinum* by ddPCR compared with q-PCR, which are important for laboratory diagnosis and epidemiology work.

## Introduction

Botulism is a life-threatening disease caused by the action of BoNTs produced by *Clostridium botulinum* (*C. botulinum*) (Weigand et al., 2015). The lethal amount of botulinum toxin in mice is 0.5~5 ng/kg, and about 1 ng/kg in humans, which is the strongest natural biological toxin known (Gill, 1982; Arnon et al., 2001). In recent years, many cases of botulism including infant botulism and food-related botulism have been diagnosed in China (Zhang et al., 2020; Lu et al., 2021; Zhu and Fu, 2021). The high mortality of botulism makes rapid diagnosis critical for treatment. Apart from the clinical symptoms and toxin exposure history, positive laboratory results are essential for clinical diagnosis. Laboratory detection of botulinum toxin and *C. botulinum* is also a growing concern due to the increasing cases of botulism in China (Xin et al., 2019). The only currently admissive standard method for detection and identification of botulinum neurotoxin is the mouse bioassays (MBAs) which cause animal ethics issue and are time-consuming (Ferreira et al., 2004). At present, the detection methods for toxin-producing species mainly include isolation and culture (CfDCaP (CDC), 2016), PCR methods (Cordoba et al., 2001; Akbulut and Grant, 2004; Heffron and Poxton, 2007; Kasai et al., 2007; Dahlsten et al., 2008; Joshy et al., 2008; Fach et al., 2009; Hill et al., 2010; Kirchner et al., 2010; Lindberg et al., 2010; Peck et al., 2010; Satterfield et al., 2010; Anniballi et al., 2013; Fohler et al., 2016; Le Marechal et al., 2018; Masters and Palmer, 2021), sequencing (Gonzalez-Escalona et al., 2018; Gonzalez-Escalona and Sharma, 2020), and matrix-assisted laser desorption ionization-time-of-flight mass spectroscopy (MALDI-TOF MS) based bacterial identification (Kalb et al., 2015; Bano et al., 2017; Xin et al., 2019; Drigo et al., 2020; Tevell Aberg et al., 2021). Most of these are time-consuming, labor-intensive, and not sensitive enough. LOD of q-PCR which is popularly used is normally between 10^1^ and 10^2^ copies in *C. botulinum* (Hill et al., 2010; Kirchner et al., 2010; Huang et al., 2019). However, clinical samples mostly contain low number of DNA molecules and below the LOD of q-PCR that can lead to false negative results by q-PCR. The highly toxic characterization of BoNT requires a more sensitive laboratory approach. Although ddPCR is widely used in many pathogens, there is no application in BoNT gene detection.

Micro-drop digital PCR is a fundamentally different method to quantifying the number of DNA compared with q-PCR (Gutierrez-Aguirre et al., 2015; Kuypers and Jerome, 2017; Maheshwari et al., 2017; Sun et al., 2018; Wang et al., 2018; Dupas et al., 2019; Capobianco et al., 2020; Cho et al., 2020; Liu et al., 2020; Xie et al., 2020; Yang et al., 2020). In ddPCR, the amplification reaction is compartmentalized into millions of independent partitions. Each partition as an individual reaction mixture contains either a single target molecule or none. The partitioned reactions are then amplified to the endpoint, which displays a positive or negative result. The absolute concentration of the target copies in the initial sample is gained from the number of positive and negative partitions (Kuypers and Jerome, 2017). Apart from the absolute quantification without reliance on a calibration curve, ddPCR has advantages not only in being less affected by sample inhibitors but also in better detection of low-copy-number samples and more precision (Morley, 2014).

Here, we aim to apply the ddPCR approach in detection of BoNT A and B genes, which are the main toxin serotypes in the clinical botulism. ddPCR assay was compared with q-PCR both in the clinical and spiked contaminated samples first revealing ddPCR assay was more sensitive than q-PCR in both *neurotoxin A* and *B* genes. Sensitivity of ddPCR was tested in 59 clinical stool samples which are positive by MBA. In total, 100% detection rate was found in ddPCR. The enrichment time for samples with low colony number of *C. botulinum* was also shortened.

## Materials and Methods

### Bacterial Strains and Plasmids

Bacterial strains and plasmids used in this study are listed in Table 1. *C. botulinum, Clostridioides difficile* (*C. difficile*), and *Clostridium perfringens* (*C. perfringens*) strains were grown anaerobically at 37 °C in TPGY media (Xin et al., 2019). *Escherichia coli* (*E. coli*), *Shigella flexneri* (*S. flexneri*), and *Shigella sonnei* (*S. sonnei*) strains were grown at 37 °C in lysogeny broth (LB) media. *Enterococcus faecium* (*E. faecium*), *Enterococcus faecalis* (*E. faecalis*), and *Listeria monocytogenes* (*L. monocytogenes*) were grown at 37 °C in brain heart infusion (BHI) broth. For accurate calculating of gene copy numbers, two plasmids containing part of BoNT A and B genes were designated as CTA PMD18-T and CTB PMD18-T with primer pair AF and AR and BF and BR, respectively (Huang et al., 2019).

**Table 1:** Strains, plasmids, and primers used in this study.

**Strains/plasmids/primers**	**Description**	**Source**
CTA PMD18-T	A 121 bp fragment containing part of toxin A gene was inserted into the vector PMD18-T	This study
CTB PMD18-T	A 130 bp fragment containing part of toxin B gene was inserted into the vector PMD18-T	This study
*C. botulinum* type A	Clinically isolated strain	This study, from a foodborne botulism in 2019 from Xinjiang Province
*C. botulinum* type B	Clinically isolated strain	This study, from an infant botulism in 2015 from Hebei Province
*C. botulinum* type E	Clinically isolated strain	This study, from a foodborne botulism in 2019 from Hebei Province
*E. faecalis*	ATCC strain	ATCC35667
*E. faecium*	Clinically isolated strain	This study
*Enterotoxigenic E. coli*	Clinically isolated strains	This study
*S. flexneri*	Clinically isolated strains	This study
*S. sonnei*	ATCC strain	ATCC25931
*C. perfringens*	Clinically isolated strains	This study
*L. monocytogenes*	Clinically isolated strains	This study
*Enteroinvasive E. coli*	Clinically isolated strains	This study
A-F	taataaaatatgggttattccagaaagag	3316560-3316589 in *C. botulinum* CDC 69094 (Huang et al., 2019)
A-R	tgttgaatcataatatgaaactggaact	3316644-3316671 in *C. botulinum* CDC 69094 (Huang et al., 2019)
A-P	5'-FAM-tcctgaagaaggagatttaaatccaccaccag-BHQ1-3'	3316602-3316633 in *C. botulinum* CDC 69094 (Huang et al., 2019)
B-F	cacaaacattgctagtgtaactgttaataa	3369988-3370017 in *C. botulinum* CDC 69094 (Huang et al., 2019)
B-R	ctatagtctcattttcatttaaaactggc	3370090-3370118 in *C. botulinum* CDC 69094 (Huang et al., 2019)
B-P	5'-JOE-cagtaatccaggagaagtggagcgaaaaaagg-BHQ2-3'	3370024-3370053 in *C. botulinum* CDC 69094 (Huang et al., 2019)

### Clinical Specimens

In total, 51 stool samples from hospitals or local Centers for Disease Control and Prevention confirmed by culture were used in this study. In total, 47 samples contain botulinum toxin B. The other seven are positive with botulinum toxin A. DNA extraction followed by qPCR and ddPCR were routinely immediately carried out after sample reception. Genomic DNA was extracted using QIAamp PowerFecal DNA Kit (catalog no. 51106; Qiagen, Germantown, MD) and stored at−20 °C until use. MBA and enrichment culture were usually performed in 1–2 days when the materials are prepared.

### Reference Testing

MBA and culture isolation of *C. botulinum* were both used as reference testing. MBA were carried out as mentioned by Wenwen Xin et al (Xin et al., 2019). Briefly, stool samples were diluted in GelPhos buffer (30 mM sodium phosphate (pH 6.2) and 0.2 % gelatin) in 1:50 and injected into 15–17 g ICR mouse intraperitoneally after centrifugation. Positive samples with classical symptoms were confirmed by antitoxins (Lanzhou Institute of Biological Products co., LTD). Stool samples were also cultured in cooked meat medium and TPGY media as described by Wenwen Xin et al. Identification was confirmed by Gram staining, MBA, and MALDI-TOF MS.

### Real-Time Quantitative PCR and Droplet Digital PCR

ddPCR is compatible with TaqMan hydrolysis probes as reported (Schaumann et al., 2018). So, same primers and probes were used for q-PCR and ddPCR (Table 1). Primer A–F, A–R, and probe A–P were used for BoNT *A* genes. Primers B–F, B–R, and probe B–P were used for BoNT B gene. q-PCR was performed as described earlier (Huang et al., 2019). If Ct value ≤ 35 is considered positive. ddPCR was carried out with QX200™ Droplet Generator, QX200™ Droplet Reader, C1000 Touch™Thermal Cycle, PX1™ PCR Plate sealer (Bio-Rad, USA), Microdrop Digital PCR Quantification Kit (Bole Corporation, USA), and ddPCR Super mix (Bio-Rad, USA). The annealing temperature and experimental components of ddPCR for *C. botulinum* types A and B gene were optimized. For both *C. botulinum* types A and B gene primers, a series of 100, 200, 300, 400, 500, 600, 700, 800, 900, and 1,000 nmol/L were tested with a probe concentration of 800 nmol/L and 60 °C for annealing temperature. A series of 100, 150, 200, 250, 300, 350, 400, and 450 nmol/L were tested for probes with selected concentration primers and 60°C for annealing temperature. Twelve gradients including 46.0, 47.1, 48.6, 50.4, 52.6, 54.8, 57.3, 59.4, 61.6, 63.4, 64.9, and 66.0 °C were examined with selected concentration of primers and probes.

### Simulation of Stool Samples

*C. botulinum* types A or B strains were inoculated into TPGY medium and incubated for 3 days in anaerobic cabinet at 37 °C. Colony-forming units (CFUs) were calculated on plates. Each 0.25 g of stool suspended in 1 ml gelatin phosphate buffer from healthy infants was added to 6 tubes containing 15 ml TPGY medium. In total, 100 μl *C. botulinum* types A or B strains of six diluted culture (from 10^6^ to 10^1^) were inoculated to 6 tubes. The blank control was added with 100 μl distilled water. Genomic DNA was extracted as described earlier. ddPCR and q-PCR were both performed in triplicate.

### Evaluation of Culture Time of Low Concentration DNA Samples in Enrichment Medium

*C. botulinum* types A and B strains were anaerobically inoculated into TPGY medium at 30 °C for 7 days until 99 % vegetative cells turn into spore-bearing vegetative sporangia. The harvested spores were washed by distilled water. The suspension was heated at 60 °C for 15 min to kill the vegetative cells. A spore suspension containing 10^6^ cfu/ml was obtained. The suspensions were serially diluted 10-fold with sterile saline. The spore numbers were calculated on plates after culture and 10 and 100 CFU/ml were used for inoculation. Since inoculation in TPGY medium, sampling for ddPCR and q-PCR was carried out every 4 h till 72 h.

## Results

### Optimization of DdPCR

**Figure 1:**
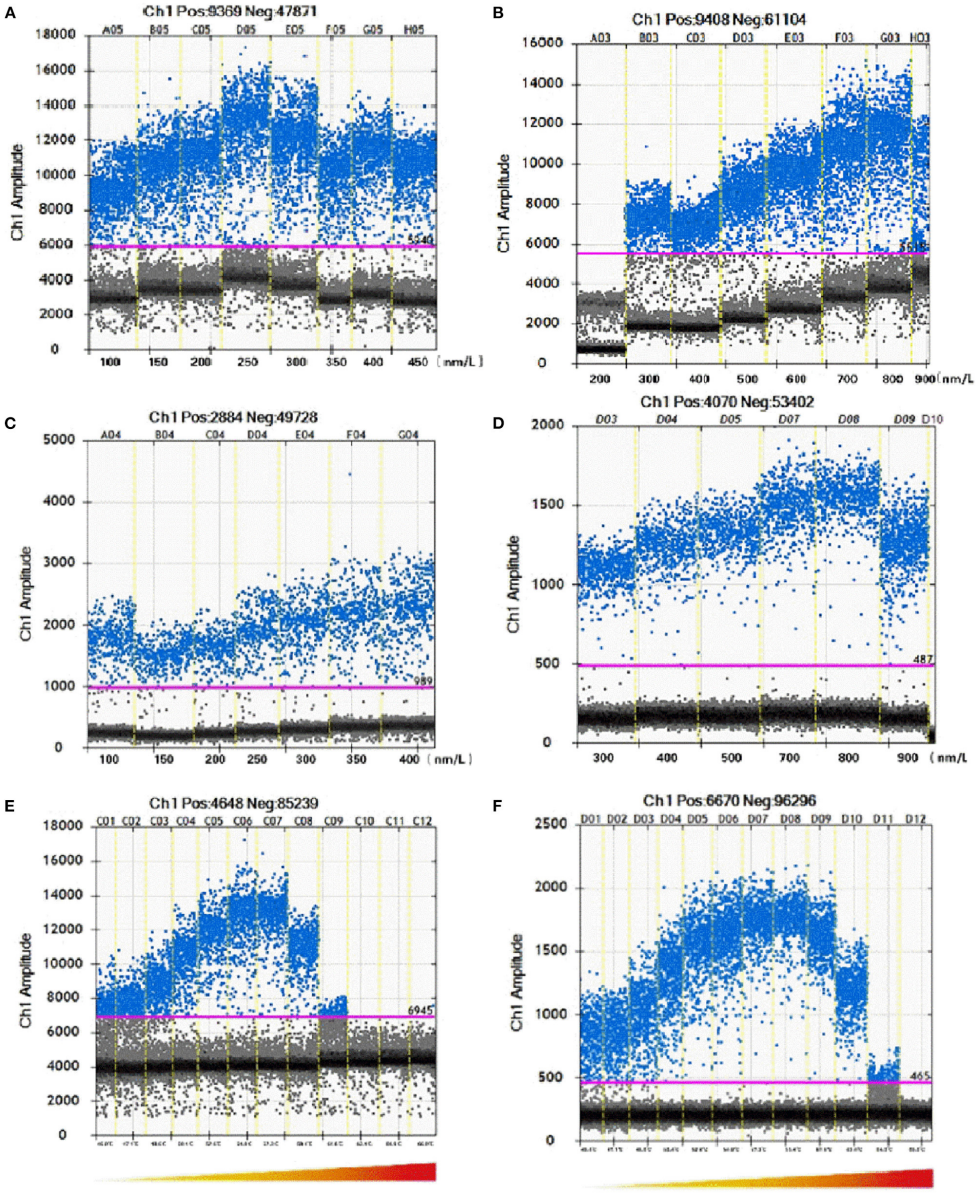
Optimization of ddPCR parameters including concentrations of primers and probes and annealing temperature. The pink line is the threshold. Blue dots represent positive droplets and gray dots represent negative droplets. **(A,B)** Demonstrated that ddPCR in *C. botulinum* serotype A with different concentrations of primers and probe. **(C,D)** Demonstrated that ddPCR in *C. botulinum* serotype B with different concentrations of primers and probes. **(E,F)** Demonstrated that ddPCR in *C. botulinum* serotypes A and B with different annealing temperature.

Each ddPCR experiment should contain at least 10,000 droplets. To optimize the primer and probe concentrations, a series of each content was set as shown in Figure 1. For *C. botulinum* type A BoNT gene amplification, the optimal concentrations of primer and probe are 900 nmol/L and 250 nmol/L, respectively (Figures 1A,B). The optimized primer and probe concentrations for *C. botulinum* type B BoNT gene are 800 nmol/L and 450 nmol/L (Figures 1C,D). With the optimized concentrations of primers and probes, reactions at 57.3 °C gave a highest positive droplet proportion (Figures 1E,F).

### Evaluation of Specificity of DdPCR and Sensitivity of q-PCR and DdPCR

**Figure 2:**
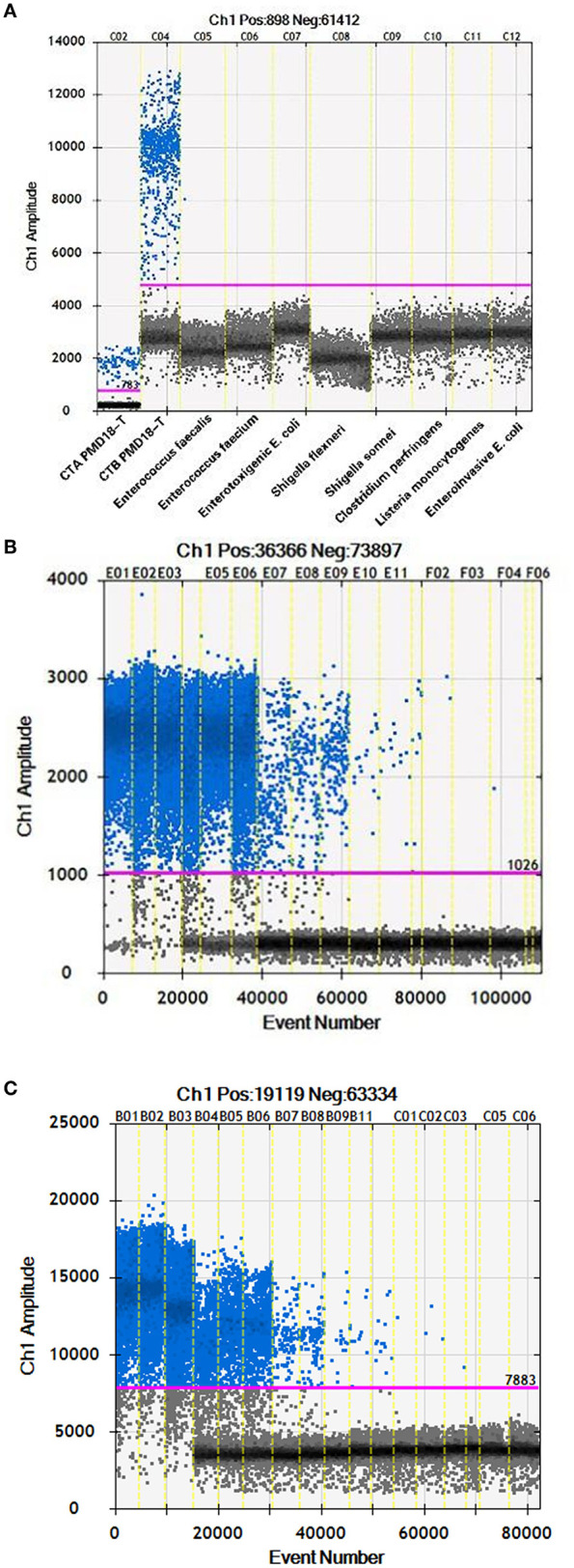
**(A)** The first two reactions were *C. botulinum* toxins A and B, respectively. **(B)** The 18 reactions were serial dilutions of CTA PMD18-T from 8.4 × 10^5^ to 8.4 × 10^−1^ with triplicate. **(C)** The 18 reactions were serial dilutions of CTB PMD18-T from 8.8×10^5^ to 8.8×10^−1^ with triplicate.

To test the specificity of ddPCR in *C. botulinum* types A and B BoNT genes, 2 *C. botulinum* and 10 other strains were used (Table 1). The target DNA from *C. botulinum* types A and B strains has been amplified successfully (Figure 2A), while no amplification was detected for the other nine control bacterial strains tested including *C. botulinum* serotype E (Figure 2A), indicating that primers and probes were specific for *C. botulinum* types A and B BoNT genes. The sensitivities of q-PCR and ddPCR were compared using constructed plasmid DNA as standard. Serial dilutions of CTA PMD18-T from 8.4 × 10^5^-8.4 × 10^−1^ and CTB PMD18-T from 8.8 × 10^5^-8.8 × 10^−1^ with triplicate were tested. The lowest concentration detected by ddPCR was 0.84 and 0.88 copies/μl for toxins A and B, respectively (Figures 2B,C). The LOD of q-PCR in serotypes A and B using same primers and probes were 5.04 × 10^2^ and 6.91 × 10^2^ copies/μl, respectively (Huang et al., 2019).

### Sensitivity in Spiked Stool and Clinical Stool Samples With q-PCR and DdPCR

Serial dilutions of *C. botulinum* types A or B strain cultures were added to normal children's stool to mimic the clinical stool samples for sensitivity evaluation. The concentrations of *C. botulinum* type A strain were 8.1 × 10^3^-8.1 × 10^0^ CFU/100 μl. The diluted concentrations of *C. botulinum* type A strain were 9.7 × 10^3^-9.7 × 10^0^ CFU/100 μl. The LOD of *C. botulinum* types A or B in spiked stool is 81 and 97 CFU/μl, respectively, by ddPCR (Tables 2, 3). Consistently, 8.1 × 10^2^ and 9.7 × 10^2^ CFU/μl was the LOD with q-PCR of *C. botulinum* types A or B, respectively (Tables 2, 3).

**Table 2:** Comparison of q-PCR and ddPCR using spiked stool samples with *C. botulinum* type A strain.

**Colony number (CFU/ul)**	**ddPCR**					**q-PCR**			
	**Number of copies (per ul specimen)**	**Average**	**CV**	**Ct**	**Average**
8.1 × 10^3^	241	256	259	252	3.8%	29.41	29.27	29.23	29.30
8.1 × 10^2^	24	22	23.1	23	4.3%	33.64	34.06	33.97	33.89
8.1 × 10	1.6	1.7	1.9	1.73	8.8%	-	-	-	
8.1	0	0	0	0		-	-	-	

**Table 3:** Comparison of q-PCR and ddPCR using spiked stool samples with *C. botulinum* type B strain.

**Colony number (CFU/ul)**	**ddPCR**					**q-PCR**		
	**Number of copies (per ul specimen)**	**Average**	**CV**	**Ct**	**Average**
9.7 × 10^3^	147	155	152	151.3	2.6%	30.25	30.33	30.30	30.29
9.7 × 10^2^	18	16	17.1	17.03	5.9%	34.89	34.83	34.76	34.83
9.7 × 10	1.41	1.5	1.43	1.45	3.3%	-	-	-	
9.7	-	-	-	-		-	-	-	

All 59 suspected clinical stool samples were tested with MBA, culture isolation, ddPCR, and q-PCR (Supplementary Table S1). In these 59 stool samples, 47 were positive by MBA with 32 isolated strains, 21 are positive by q-PCR and 49 are positive by ddPCR. Among them, four stools were identified toxin A, isolated serotype A strains and confirmed by ddPCR with none was detected by q-PCR. In total, 43 stool samples were detected with toxin B by MBA in which 28 serotype B strains were isolated, 19 were positive by q-PCR and 45 were verified by ddPCR. Interestingly, two stools which are negative by MBA were detected by ddPCR.

### Repeatability Verification

The intra-batch reproducibility experiment is to repeat the same sample in the same reaction system for three times. The results showed that the number of positive droplets between the reactions of the same concentration template is similar, and the coefficient of variation of the botulinum toxin type A plasmid is 4.2% and 2.4%, 5.9%; the coefficient of variation of botulinum toxin type B plasmids were 4.5%, 4.9%, and 5.4%, both of which were <6%. It showed that the established ddPCR detection system has good repeatability.

### Culture Time of Low Concentration DNA Samples in Enrichment Medium

To test the enrichment time of low concentration DNA samples which are below the LOD with q-PCR, two samples containing 10 and 100 spores were cultured in TPGYT medium. In total, 44 and 28 h were the shortest enrichment time for samples containing 10 and 100 spores of *C. botulinum* type A, respectively, by ddPCR (Tables 4, 5). Consistently, the shortest enrichment time were 48 and 32 h by q-PCR. For *C. botulinum* type B spores, 48 and 32 h were required for detection by ddPCR (Tables 4, 5) for 10 and 100 spores, respectively. Similarly, 52 and 36 h enrichment time were at least required by q-PCR.

**Table 4:** The growth of serotypes A and B strains in enrichment culture with 10 spores inoculation detected by ddPCR and q-PCR.

**Culture time (h)**	**44**	**48**	**52**	**56**	**60**	**64**	**68**
Serotype A	1.4	10.2	54	266	4,486	6,845	7,047
by ddPCR	1.3	11	56	251	4,521	6,914	7,086
(Copies/μl)	1.6	12	60	271	4,398	6,628	7,035
CV	10.7%	8.2%	5.4%	4.0%	1.4%	2.2%	0.4%
Serotype A		34.36	30.17	26.25	21.03	17.42	13.39
by q-PCR	-	34.77	30.26	26.38	21.35	17.51	13.22
(Ct value)	-	35.04	30.10	26.13	21.24	17.23	13.44
CV	-	1.0%	0.3%	0.5%	0.8%	0.8%	0.9%
Serotype B	-	1.2	4.5	19.2	174	6,856	-
by ddPCR	-	1.1	4.6	19.4	185	6,921	-
(Copies/μl)	-	1.4	4.9	19.7	192	6,847	-
CV	-	12.4%	4.5%	1.3%	4.9%	0.6%	-
Serotype B	-	-	35.27	28.25	25.43	21.42	-
by q-PCR	-	-	35.06	28.38	25.35	21.51	-
(Ct value)		-	35.34	28.13	25.24	21.23	
CV			0.4%	0.4%	0.4%	0.7%	

**Table 5:** The growth of serotypes A and B strains in enrichment culture with 100 spores inoculation detected by ddPCR and q-PCR.

**Culture Time(h)**	**28**	**32**	**36**	**40**	**44**	**48**	**52**
Serotype A	2.7	28	150	3,640	6,052	7,082	7,049
by ddPCR	2.6	25.3	162	3,570	6,668	7,017	7,026
(Copies/μl)	2.9	27	173	3,527	6,324	7,074	7,033
CV	5.6%	5.1%	7.1%	1.6%	4.9%	0.5%	0.2%
Serotype A	-	32.98	27.86	22.12	18.11	12.14	10.08
by q-PCR	-	32.80	27.63	21.92	18.12	12.34	10.11
(Ct value)	-	61.96	27.76	21.89	18.11	12.18	10.15
CV	-	39.4%	0.4%	0.6%	0.03%	0.9%	0.4%
Serotype B	-	1.6	7.6	30	330	7,082	-
by ddPCR	-	1.7	8.7	33	342	7,017	-
(Copies/μl)	-	1.8	7.5	35	350	7.34	-
CV	-	5.9%	8.4%	7.7%	3.0%	86.5%	
Serotype B	-	-	34.30	27.23	24.70	19.96	
by q-PCR		-	34.28	27.70	24.55	20.19	
(Ct value)		-	34.95	27.89	24.38	20.22	
CV			1.1%	1.2%	0.7%	0.7%	

## Discussion

*C. botulinum* serotypes A and B are the two main serotype causing botulism in China. As the difficulty in diagnosis, many cases of botulism are misdiagnosed which can be life-threatening (Arnon et al., 2001; CfDCaP (CDC), 2016). So, it is critically important to develop a rapid and sensitive method for the detection of botulinum toxin or BoNT-producing bacteria. Many methods have been developed to detect *C. botulinum*. MALDI-TOF MS-based bacterial identification is a rapid method providing robust accuracy in *C. botulinum* identification (Fenicia et al., 2007; Xin et al., 2019). However, MALDI-TOF MS-based bacterial identification requires enrichment and isolation of bacteria, which commonly takes days to complete, and this method cannot identify all BoNT-producing species or discriminate them from the related species (Xin et al., 2019). High-throughput sequencing and single molecule real-time sequencing could provide excellent phylogenetic information for typing and tracing the source of *C. botulinum*. Nevertheless, they are not used widely in clinical due to the requirements of highly trained staff and expensive instruments. q-PCR of toxin genes can serotype strains of *C. botulinum* and are widely used in clinical settings due to robustness, low cost, and simplicity. Kirchner *et al*. reported an LOD of 7–287 genomes by q-PCR in BoNT *A*–*F* in 95% possibilities (Kirchner et al., 2010). However, the detection probability in single PCR raised above 10 times in 100% possibilities. Fenicia *et al*. showed a LOD of 60 copies of C. botulinum type A by SYBR green real-time PCR (Barker et al., 2016). The other researchers demonstrated an LOD between 16–200 copies for BoNT gene by q-PCR (Akbulut and Grant, 2004; Kasai et al., 2007; Fach et al., 2009; Takahashi et al., 2010; Malakar et al., 2013). A minimum of 100 copies BoNT Agene was detected in spiked rice (Sedlak et al., 2014). In the laboratory, stool samples are more likely to produce a higher positive rate than serum. The gene copy number in the clinical stool DNA samples very likely below the LOD of q-PCR. Furthermore, inhibitors in stool samples can affect q-PCR efficiency (Mazaika and Homsy, 2014; Morley, 2014; Wang et al., 2016). ddPCR is an assay that combines state-of-the-art microfluidics technology with TaqMan-based PCR to achieve precise target DNA quantification at high levels of sensitivity and specificity. Because quantification is achieved without the need for standard assays in an easy to interpret, unambiguous digital readout, ddPCR is far simpler, faster, and less error prone than real-time qPCR (Mazaika and Homsy, 2014). In this study, we evaluated ddPCR in detection of *C. botulinum* toxin A and toxin B genes by comparing with q-PCR. It is seen that ddPCR not only can raise the sensitivity and shorten the enrichment time with high specificity, but also can increase the positive rate of spiked stool samples and clinical stool samples.

With optimized ddPCR, the sensitivity was increased by nearly 100 times with high specificity in constructed plasmids containing *C. botulinum* toxins A and B genes by comparing with q-PCR. In addition, the LOD was increased in spiked stool samples both in toxins A and B genes by ddPCR. This sensitivity increase remained by comparing with q-PCR and was quite critical in the clinical stool samples as proved. All 47 clinical stool samples were detected by MBA assay and were confirmed by ddPCR. Two suspected stool samples negative with MBA were identified by ddPCR indicating that ddPCR was more sensitive than MBA or none active toxin was absent in the two samples whereas toxin genes were present. Another possibility is the false positive or potential contamination in ddPCR in these two stool samples. However, as blank controls were included in every trial, this possibility is very low. Only 32 strains were isolated from 32 stool samples showing a low-isolation ratio or no live spores were existed in the other samples. Interestingly, only 21 samples were positive by qPCR suggesting a low-positive rate. This might due to the low sensitivity of qPCR and inhibitors factors in stool samples (Mazaika and Homsy, 2014; Morley, 2014; Wang et al., 2016). These results indicated that ddPCR can be used as a potential alternative diagnostic method for MBA.

Enrichment time for low colony number samples were evaluated here. Briefly, the earliest detection time was shortened by 4 h both in 10 and 100 spores samples in *C. botulinum* serotypes A and B strains with ddPCR. By sampling every 4 h since inoculation, samples containing 10 and 100 *C. botulinum* serotype A spores can be detected as early as 44 and 28 h, respectively, by ddPCR. This detection time was delayed by 4 h in *C. botulinum* serotype B spores. This indicated that growth rate differs in *C. botulinum* serotypes A and B strains with low-colony samples. An important consideration is the use of ddPCR in the isolation of *C. botulinum*. The good aspect is the sensitivity in the earlier detection from enrichment culture. As most enrichment cultures will be tested by qPCR or ddPCR and only positive cultures will be streaked in the plate. ddPCR shows the potential ability in detecting samples with few cells by its sensitivity. However, lack of DNA standards hinders the application of ddPCR in the clinical and laboratory tests.

In conclusion, here dd-PCR was demonstrated it can be used as a more accurate detection method in the clinical diagnosis by increasing sensitivity in stool samples and culture isolation by shortening enrichment time.

## Data Availability Statement

The original contributions presented in the study are included in the article/Supplementary Material, further inquiries can be directed to the corresponding authors.

## Ethics Statement

The animal study was reviewed and approved by Laboratory Animal Welfare and Ethics Committee in IVDC, China CDC.

## Author Contributions

PG, CW, JZ, SW, YH, XX, and WX performed the experiments, analyzed the data, and wrote the manuscript. XX and WX conceptualized and designed the study. YD, TL, and CY provided material and samples. All authors reviewed and edited the manuscript and read and approved the final manuscript.

## Funding

This work was supported by the National Key Research and Development Program of China (No. 2018YFC1603800).

## Conflict of Interest

The authors declare that the research was conducted in the absence of any commercial or financial relationships that could be construed as a potential conflict of interest. The reviewer JH declared a shared affiliation with several of the authors, YH, CY, and XX, to the handling editor at time of review.

## Publisher's Note

All claims expressed in this article are solely those of the authors and do not necessarily represent those of their affiliated organizations, or those of the publisher, the editors and the reviewers. Any product that may be evaluated in this article, or claim that may be made by its manufacturer, is not guaranteed or endorsed by the publisher.
