# Supplementary material for: Evaluation and Optimization of Microdrop Digital PCR for Detection of Serotype A and B Clostridium botulinum
Source: Front Microbiol. 2022 May 9;13:860992. doi: 10.3389/fmicb.2022.860992 (PMC9125207; doi:10.3389/fmicb.2022.860992)
Supplement: Supplementary file 1 [file Data_Sheet_1.PDF]

Table S1 Comparison of the MBA, culture isolation q-PCR and ddPCR

Sample ID	Source	BoNTs by MBA	Isolates serotype	q-PCR	ddPCR
1505271-1	Beijing	Neg	NT	NEG	NEG
1505292-1	Hebei	B	B	NEG	POS
1505292-2	Hebei	B	B	NEG	POS
1505292-3	Hebei	B	B	NEG	POS
1505292-4	Hebei	B	B	NEG	POS
1505293-1	Hebei	B	B	NEG	POS
1607214-1	Xinjiang	A	A	NEG	POS
1607214-2	Xinjiang	A	A	NEG	POS
1805165-1	Beijing	B	B	POS	POS
1806086-1	Hebei	B	B	POS	POS
1806086-2	Hebei	B	B	NEG	POS
1811217-1	Shandong	B	B	POS	POS
1811217-2	Shandong	B	B	NEG	POS
1902178-1	Hebei	B	B	POS	POS
1904158-2	Hebei	B	B	NEG	POS
1902189-1	Beijing	B	B	POS	POS
1904129-2	Beijing	B	B	POS	POS
1905119-3	Beijing	B	B	NEG	POS
1906129-4	Beijing	B	B	NEG	POS
1906159-5	Beijing	NEG	NEG	NEG	NEG
19080111-1	Inner Mongolia	B	B	POS	POS
19080711-2	Inner Mongolia	B	B	POS	POS
19080911-3	Inner Mongolia	B	B	POS	POS
19081311-4	Inner Mongolia	B	B	NEG	POS
19081511-5	Inner Mongolia	B	NEG	NEG	POS
19081911-6	Inner Mongolia	NEG	NEG	NEG	NEG
19101012-1	Shanxi	B	B	POS	POS

Sample ID	Source	BoNTs by MBA	Isolates serotype	q-PCR	ddPCR
19101512-2	Shanxi	B	B	POS	POS
19112012-4	Shanxi	NEG	NEG	NEG	POS
19110113-1	Hebei	NEG	NEG	NEG	NEG
19111514-1	Hubei	NEG	NEG	NEG	NEG
19112115-1	Inner Mongolia	B	B	NEG	POS
19111216-1	Hunan	A	A	POS	POS
19101016-2	Hunan	A	A	POS	POS
19041717-1	Beijing	B	B	POS	POS
19051317-2	Beijing	B	NT	POS	POS
19051617-3	Beijing	B	NT	POS	POS
19051817-4	Beijing	B	NT	POS	POS
19051917-5	Beijing	B	NT	POS	POS
19052217-7	Beijing	B	NT	NEG	POS
19052217-8	Beijing	B	NT	NEG	POS
19052317-9	Beijing	B	NT	NEG	POS
19052317-10	Beijing	B	NT	NEG	POS
19052417-11	Beijing	B	NT	NEG	POS
19052517-12	Beijing	B	NT	NEG	POS
19052617-13	Beijing	B	NT	NEG	POS
19052817-14	Beijing	NEG	NT	NEG	POS
19010617-15	Beijing	NEG	NT	NEG	NEG
20061718-1	Hebei	NEG	NEG	NEG	NEG
20070919-1	Hebei	NEG	NEG	NEG	NEG
20080320-2	Beijing	B	B	POS	POS
20080420-3	Beijing	B	B	POS	POS
20080620-4	Beijing	B	B	NEG	POS
20080720-5	Beijing	B	NEG	NEG	POS
20081220-6	Beijing	NEG	NT	NEG	NEG

Sample ID	Source	BoNTs by MBA	Isolates serotype	q-PCR	ddPCR
20081722-1	Inner Mongolia	NEG	NEG	NEG	NEG
20092123-1	Hebei	B	NEG	POS	POS
20100923-2	Hebei	B	NEG	NEG	POS
20103024-1	Hebei	B	B	NEG	POS

NEG: Negative; NT: not tested; B: neurotoxin B; POS: positive; A: neurotoxin A.
